# Supplementary material for: Regulation of mammalian 3D genome organization and histone H3K9 dimethylation by H3K9 methyltransferases
Source: Commun Biol. 2021 May 13;4:571. doi: 10.1038/s42003-021-02089-y (PMC8119675; doi:10.1038/s42003-021-02089-y)
Supplement: Supplementary file 2 — Supplementary Information [file 42003_2021_2089_MOESM2_ESM.pdf]

Regulation of mammalian 3D genome organization and histone H3K9 dimethylation by H3K9 methyltransferases

Kei Fukuda, Chikako Shimura, Hisashi Miura, Akie Tanigawa, Takehiro Suzuki, Naoshi Dohmae, Ichiro Hiratani and Yoichi Shinkai

Supplementary Materials

Contents

Supplementary Fig. 1 Characterization of SETDB1 and SUV39H1/2 dependent H3K9me2 region in mESCs..2

Supplementary Fig. 2 Characterization of SETDB1 and SUV39H1/2 dependent H3K9me2 region in iMEFs…5

Supplementary Fig. 3 Recovery of H3K9me2 after the UNC0642 treatment……………………………………………7

Supplementary Fig. 4 Function of G9a/GLP-independent H3K9me2 in transcriptional regulation……………..8

Supplementary Fig. 5 Correlation of decreased H3K9me2 and active compartment formation…………………9

Western blotting data ………...12

Supplementary Table 1 Correlation between replicate in RNA-seq, ChIP-seq, Hi-C seq…………………………………14

Supplementary Table 2 Primer list for ChIP-qPCR. qRT-PCR and genotyping…………………………………………………15

Supplementary Table 3 Oligonucleotide for gRNA vector construction……………………………………………………16

Supplementary Table 4 Sumary of NGS data set……………………………………………………………………………………17

Supplementary Fig. 1

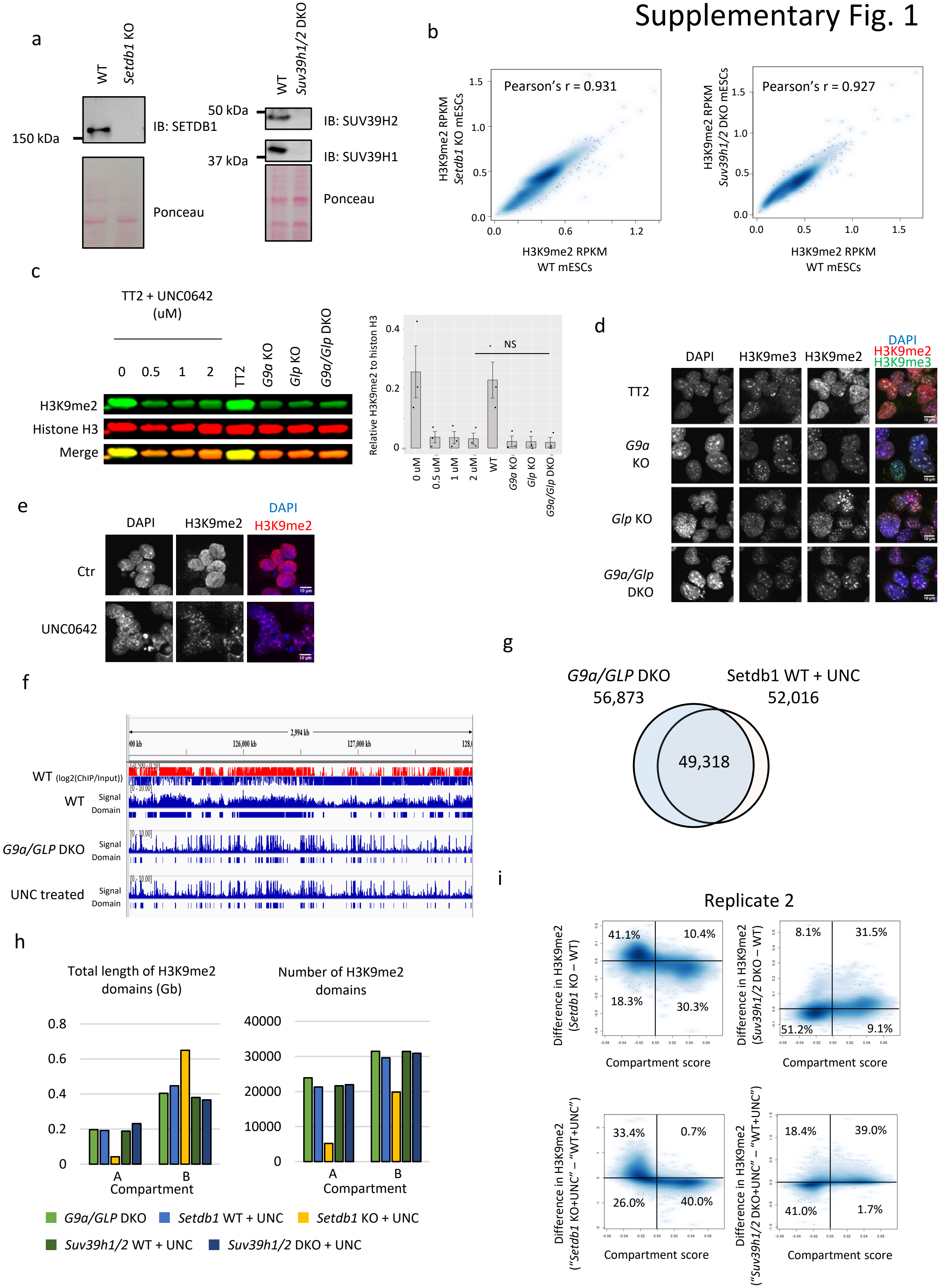

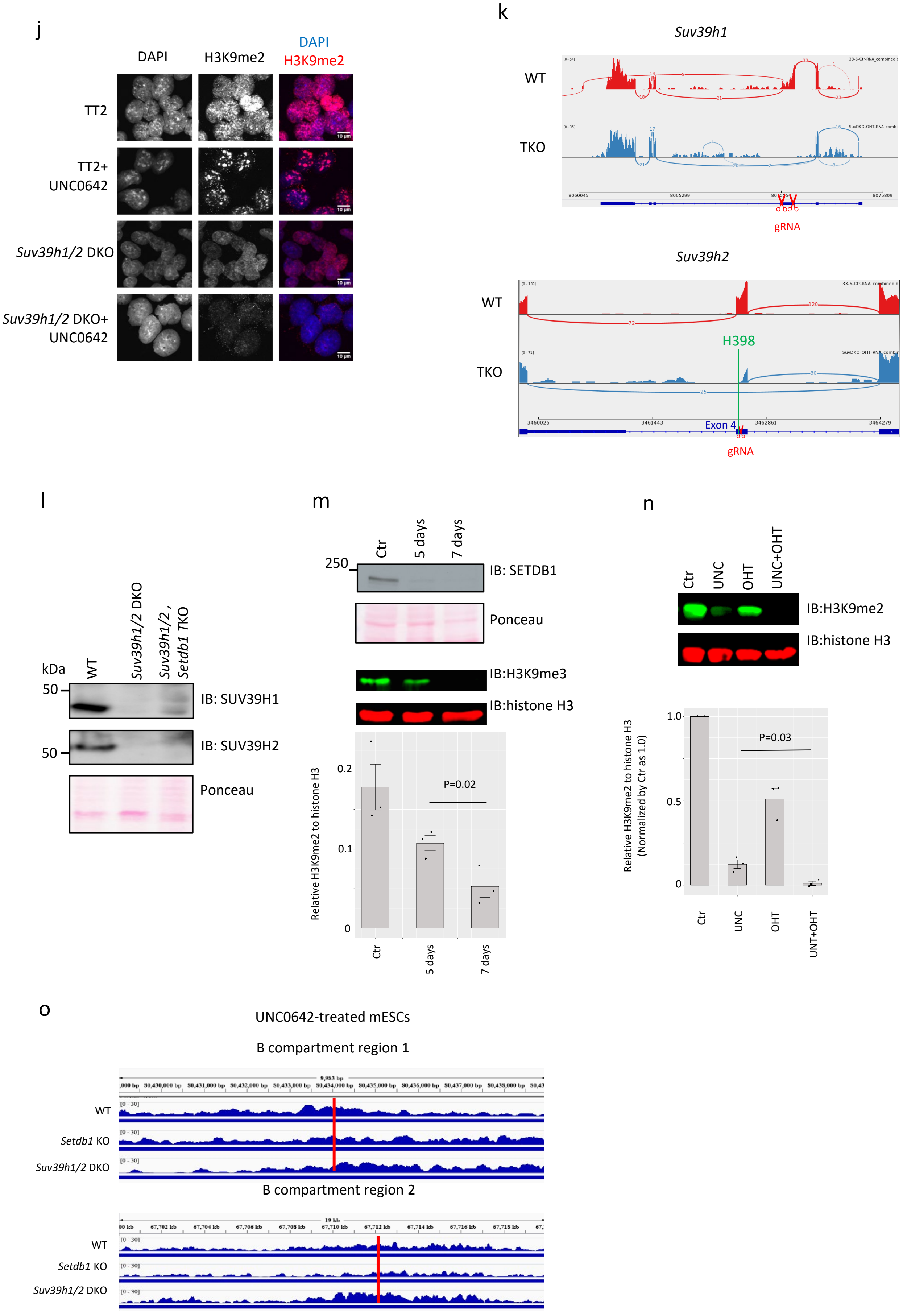
j
DAPI
H3K9me2
DAPI
H3K9me2
TT2
TT2+
UNC0642
Suv39h1/2 DKO
Suv39h1/2 DKO+
UNC0642
10 µm
k
Suv39h1
WT
TKO
gRNA
Suv39h2
WT
H398
TKO
Exon 4
gRNA
l
kDa
WT
Suv39h1/2 DKO
Suv39h1/2 , Setdb1 TKO
50
IB: SUV39H1
50
IB: SUV39H2
Ponceau
m
Ctr
5 days
7 days
250
IB: SETDB1
Ponceau
IB:H3K9me3
IB:histone H3
Relative H3K9me2 to histone H3
0.2
0.1
0
P=0.02
Ctr
5 days
7 days
n
Ctr
UNC
OHT
UNC+OHT
IB:H3K9me2
IB:histone H3
Relative H3K9me2 to histone H3
(Normalized by Ctr as 1.0)
1.0
0.5
0
P=0.03
Ctr
UNC
OHT
UNT+OHT
o
UNC0642-treated mESCs
B compartment region 1
WT
Setdb1 KO
Suv39h1/2 DKO
B compartment region 2
WT
Setdb1 KO
Suv39h1/2 DKO

Supplementary Fig. 1

a Validation of *Setdb1* KO and *Suv39h1/2* DKO mESCs by western blotting. SETDB1 protein is not detectable five days after 4-OHT treatment in *Setdb1* cKO mESCs. **b** Comparison of H3K9me2 RPKM in 80-kb bins between before and after OHT-treatment of *Setdb1 c*KO mESCs (#33-6)[8] (WT vs *Setdb1* KO) or WT and *Suv39huv39h1/2* KO mESCs. H3K9me2 profiles are not changed largely in *Setdb1* or *Suv39h1/2* KO mESCs. Darker blue represents higher dot density. **c** Western blotting analysis of H3K9me2 in *G9a/GLP* KO mESCs and UNC0642-treated mESCs. UNC0642 treatment for three days reduces H3K9me2 to levels comparable to *G9a* and/or *GLP* KO mESCs. . Bar graph shows amounts of H3K9me2 relative to histone H3. Data are mean ± SEM; *n* = 3. NS: P>0.05. Statistics comparison was only shown between TT2 (TT2) treated with 2uM of UNC0642 and G9a/GLP DKO KO mESCs. P-value was calculated by paired Student's t-test. **d** Immunofluorescence analysis of H3K9me2/3 in *G9a/GLP* KO mESCs. While H3K9me2 is localized in the entire nucleoplasm in WT mESCs, it is enriched in DAPI-dense loci in *G9a/GLP* KO mESCs. IF analysis was performed for 2 times. All scale bars represent 10 µm. **e** Immunofluorescence analysis of H3K9me2 in UNC0642-treated mESCs. Consistent with *G9a/GLP* KO mESCs, H3K9me2 is enriched in DAPI-dense loci in UNC0642-treated mESCs. IF analysis was performed for 2 times. All scale bars represent 10 µm. **f** A representative view of H3K9me2 ChIP-seq data in WT, *G9a/GLP* DKO and UNC0642-treated mESCs. Blue boxes represent H3K9me2 domains identified by *Hiddendomains*. The size of H3K9me2 domains in *G9a/GLP* or UNC0642-treated mESCs are smaller than those in WT mESCs. **g** An overlap of H3K9me2 domains between *G9a/GLP* DKO mESCs and UNC0642-treated mESCs. 94.8% of H3K9me2 domains in UNC0642-treated mESCs is overlapped with those in *G9a/GLP* DKO mESCs. **h** The length and the number of H3K9me2 domains in *G9a/GLP* DKO or UNC0642-treated mESCs. In UNC0642-treated *Setdb1* KO mESCs, both the total length and the number of H3K9me2 domains in the A compartments are decreased. **i** Scatter plot of compartment score in WT mESCs versus changes of H3K9me2 RPKM in *Setdb1* or *Suv39h1/2* DKO mESCs treated with or without UNC0642 from biological replicate data. **j** Immunofluorescence analysis of H3K9me2 in UNC0642-treated *Suv39h1/2* DKO mESCs. H3K9me2 enrichment in DAPI-dense loci observed in UNC0642-treated mESCs is lost in UNC0642-treated *Suv39h1/2* DKO mESCs. IF analysis was performed for 2 times. All scale bars represent 10 µm. **k** Validation of *Suv39h1* and *2* mutation in TKO mESCs by RNA-seq analysis. Sashimi-plot was generated by Integrative Genome Viewer. Exon 3 of *Suv39h1* is completely deleted in TKO mESCs. *Suv39h1* is X-linked gene and one copy in the TKO mESC (male). H398 of SUV39H2, which is an essential for the methyltransferase activity, is removed from the *Suv39h2* transcripts. **l** Validation of *Suv39h1/2* KO in *Setdb1/Suv39h1/2* TKO mESCs by western blotting. **m** Validation of *Setdb1* KO and H3K9me3 loss by western blotting in TKO mESCs. five days after 4-OHT treatment is not enough to SETDB1 and H3K9me3 depletion. SETDB1 and H3K9me3 are not detectable seven days after 4-OHT treatment. Bar graph at the bottom shows amounts of H3K9me3 relative to histone H3. Data are mean ± SEM; *n* = 3. Statistics comparison was only shown between *Setdb1* cKO mESCs treated with 4-OHT for five days and seven days. P-value was calculated by paired Student's t-test. **n** Validation of H3K9me2 loss in *Setdb1/Suv39h1/2* TKO mESCs by western blotting. H3K9me2 is not detectable in UNC0642-treated TKO mESCs 7 days after 4-OHT treatment. Bar graph represents relative H3K9me2 to histone H3, normalized by control sample as 1.0. Data are mean ± SEM; *n* = 3. P-value was calculated by paired Student's t-test. **o** Representative B compartment regions in which H3K9me2 remains in both *Setdb1* KO and *Suv39h1/2* DKO mESCs treated with UNC0642. Red bars indicate a region analyzed by ChIP-qPCR in Figs. 1m and 2g.

Supplementary Fig. 2

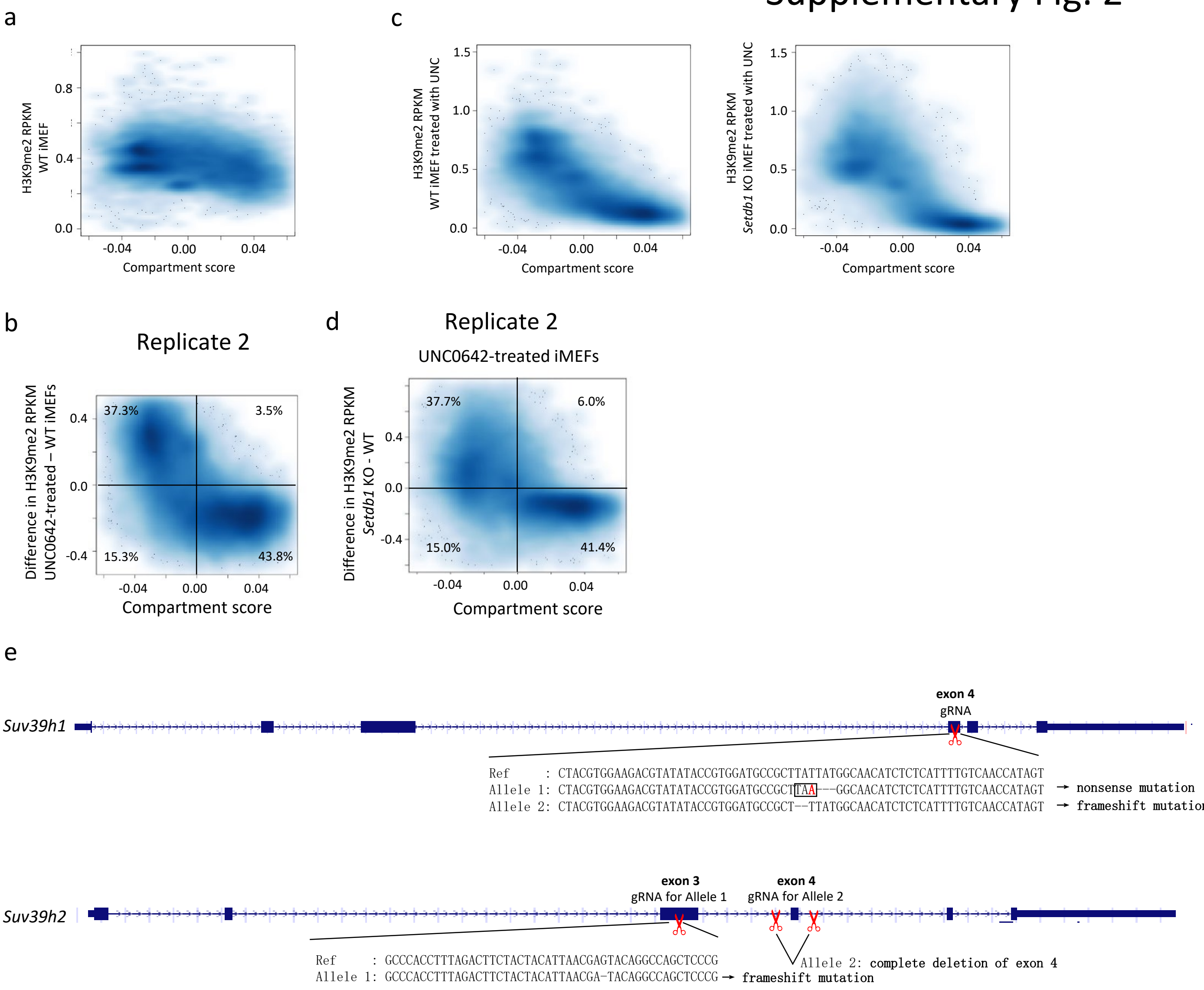

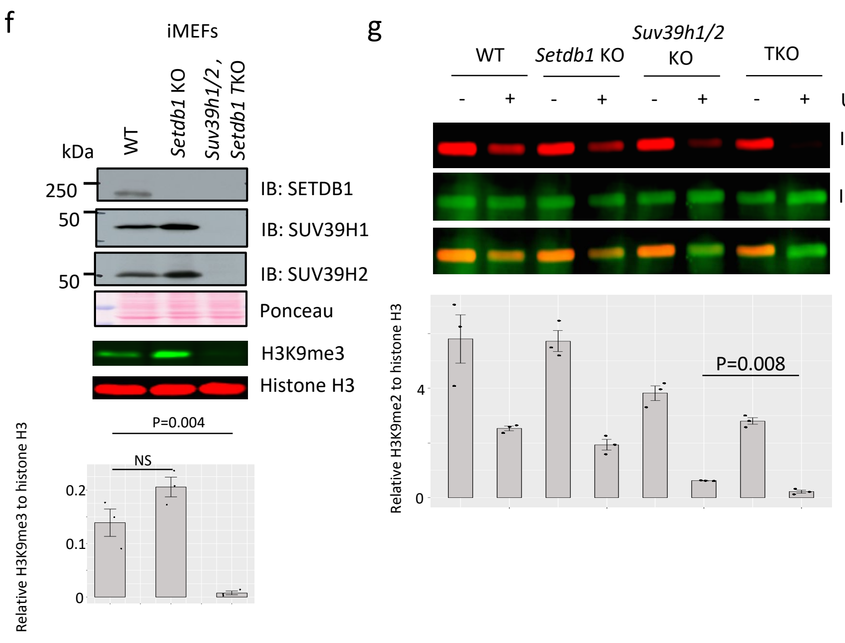

h

Relative to WT	WT+UNC	TKO	TKO+UNC
H3K9me1	44%	188%	14%
H3K9me2	24%	30%	1%
H3K9me3	35%	1%	0%

Supplementary Fig. 2

a A scatter plot between compartment score and H3K9me2 RPKM in iMEFs. Compartment score in mESCs was used for the analysis. Each plot represents data from 80-kb bin . Darker blue represents higher dot density. **b** A scatter plot of compartment score in WT iMEFs versus difference in H3K9me2 RPKM between WT and UNC0642-treated iMEFs from biological replicate data. **c** Scatter plots of compartment score in WT iMEFs versus H3K9me2 RPKM in WT and *Setdb1* KO iMEFs treated with UNC0642. Lower H3K9me2 in the A compartments (positive value region) in UNC0642-treated *Setdb1* KO iMEFs than in UNC0642-treated WT iMEFs is observed. **d** A scatter plot of compartment score in WT iMEFs versus difference in H3K9me2 RPKM between UNC0642-treated WT and *Setdb1* KO iMEFs from biological replicate data. **e** Genotype of *Setdb1/Suv39h1/2* TKO iMEFs, which is derived from female mice. **f** Validation of *Setdb1/Suv39h1/2* TKO iMEFs by western blotting. H3K9me3 is not detectable in TKO iMEFs. Bar graph at the bottom shows amounts of H3K9me3 relative to Histone H3. Data are mean ± SEM; *n* = 3. P-value was calculated by Dunnett's multiple comparison test. NS: P>0.05. **g** Western blotting analysis of H3K9me2 in TKO iMEFs. H3K9me2 is not detectable in UNC0642-treated TKO iMEFs. Bar graph at the bottom shows amounts of H3K9me2 relative to Histone H3. Compared to UNC0642-treated *Suv39h1/2* DKO iMEFs, H3K9me2 in UNC0642-treated TKO iMEFs was significantly decreased. Data are mean ± SEM; *n* = 3. Statistics comparison was only shown between UNC0642-treated *Suv39h1/2* DKO and TKO iMEFs. P-value was calculated by paired Student’s t-test. **h** A mass spectrometry analysis of H3K9 methylation in i MEFs. iMEFs were treated with 2μM of UNC0642 for five days. Relative amounts of H3K9 methylation to WT are shown. B oth H3K9me2 and H3K9me3 were essentially lost in UNC0642-treated TKO iMEFs.

Supplementary Fig. 3

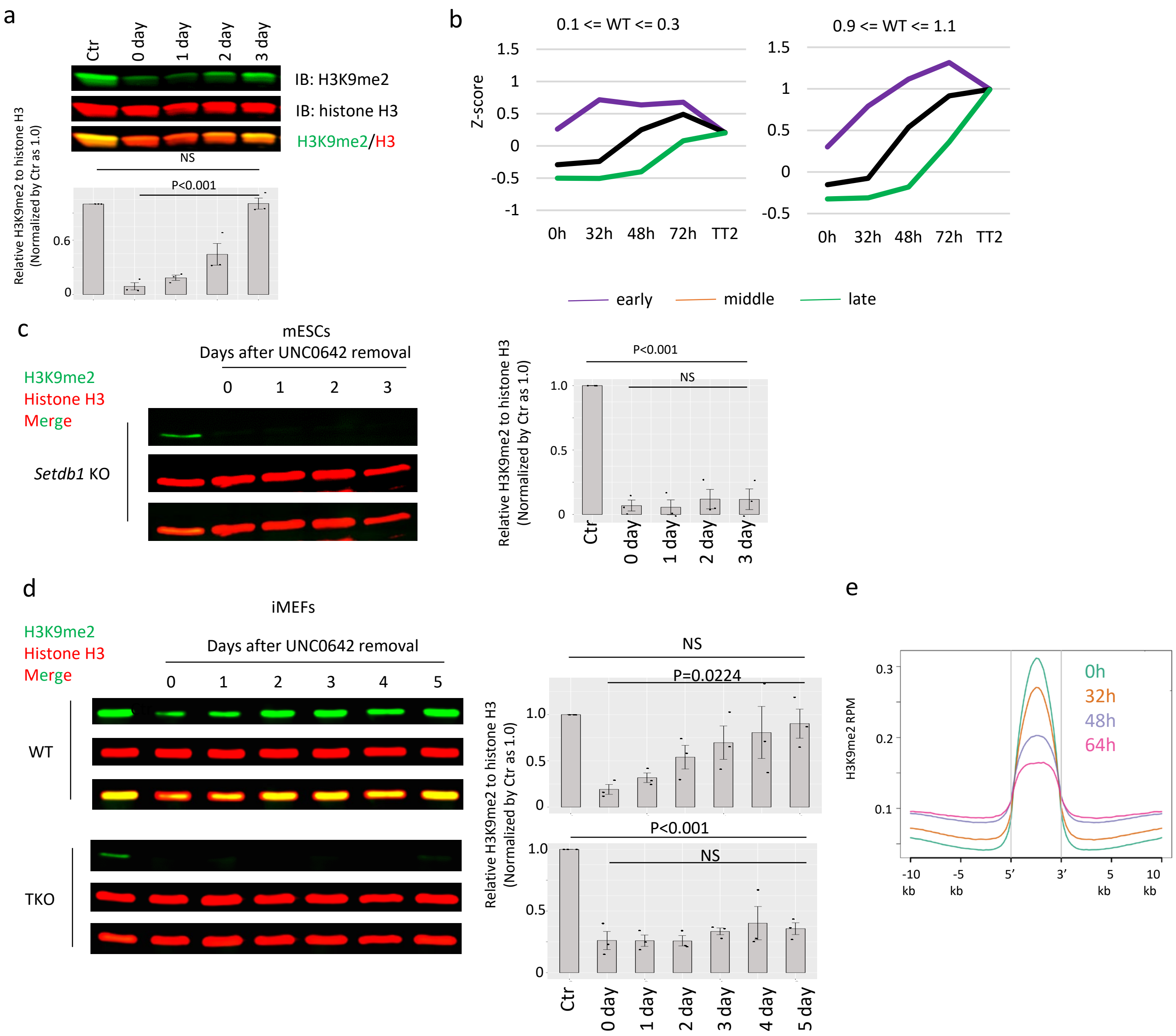

Supplementary Fig. 3

a H3K9me2 recovery after the UNC0642 treatment analyzed by western blotting. H3K9me2 is almost recovered three days after UNC0642 removal. Bar graph represents relative H3K9me2 to histone H3, normalized by control sample as 1.0. Data are mean ± SEM; *n* = 3. NS: *P* > 0.05, **P* < 0.05 by Dunnett's multiple comparison test. Statistics comparison was only shown between 3 days after UNC0642 removal and control or UNC0642-treated mESCs. **b** H3K9me2 recovery in each class. The left and right graphs use regions with Z-scores between 0.1 and 0.3 and between 0.9 and 1.1 in WT mESCs, respectively. In both cases, the regions that show faster H3K9me2 recovery tend to harbor a higher H3K9me2 level after the UNC0642 treatment. **c** H3K9me2 recovery after the UNC0642 treatment in *Setdb1* KO mESCs analyzed by western blotting. No clear H3K9me2 recovery is observed in *Setdb1* KO mESCs. Bar graph represents relative H3K9me2 to histone H3, normalized by control sample as 1.0. Data are mean ± SEM; *n* = 3. NS: *P* > 0.05 by Dunnett's multiple comparison test. Statistics comparison was only shown between 5 days after UNC0642 removal and control or UNC0642-treated iMEFs. **d** H3K9me2 recovery after UNC0642 treatment in TKO iMEFs analyzed by western blotting. No clear H3K9me2 recovery is observed in TKO iMEFs. Bar graph represents relative H3K9me2 to histone H3, normalized by control sample as 1.0. Data are mean ± SEM; *n* = 3. NS: *P* > 0.05, by Dunnett's multiple comparison test. Statistics comparison was only shown between 5 days after UNC0642 removal and control or UNC0642-treated iMEFs. **e** Enrichment of H3K9me2 around G9a/GLP-independent H3K9me2 domain after UNC0642 removal.

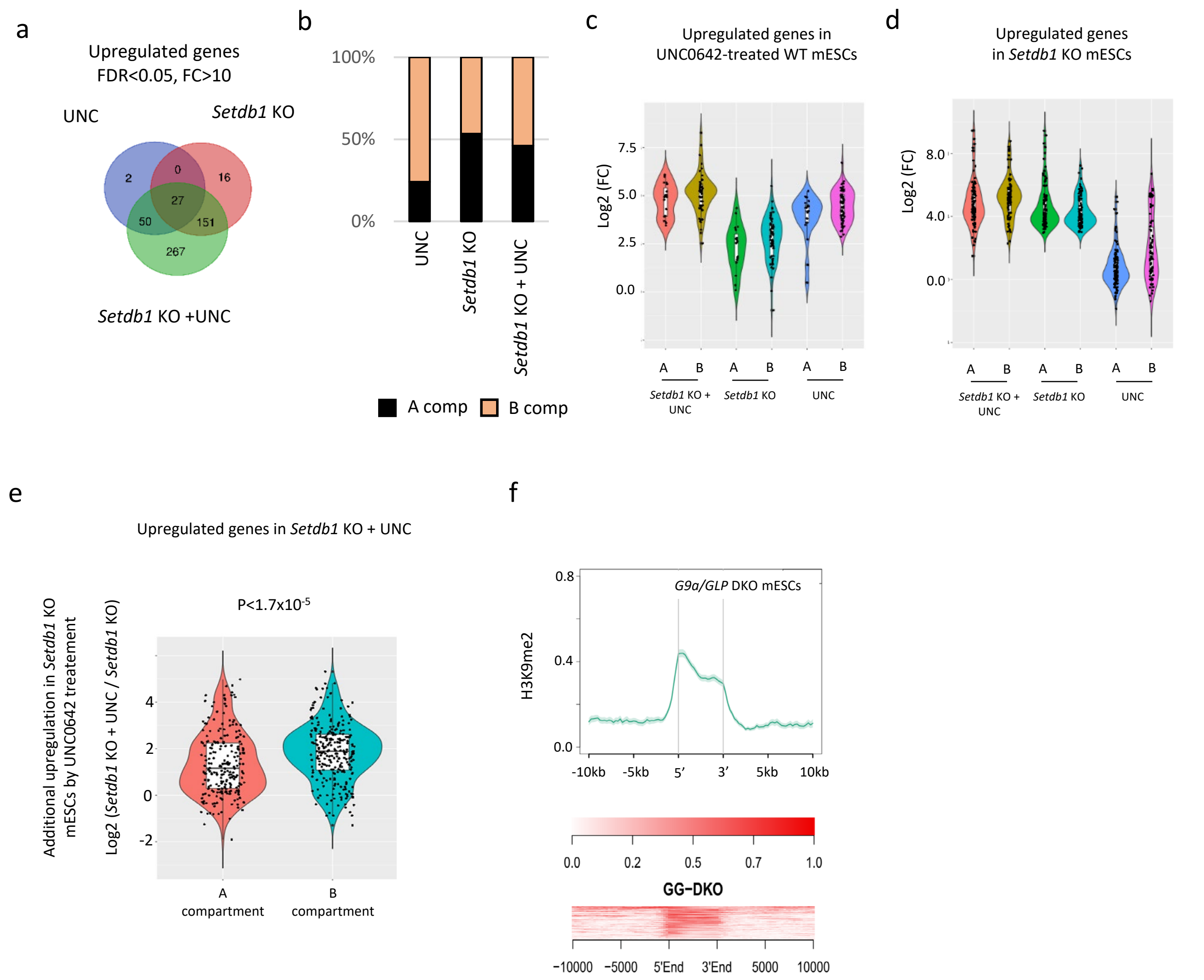

Supplementary Fig. 4

a Venn diagram of upregulated genes in each condition. **b** Distribution of upregulated genes in the A/B compartment. Majority of upregulated genes in UNC0642-treated mESCs are in the B compartments. **c** Violin plot of log2 fold changes of genes upregulated in UNC0642-treated mESCs. Genes in the A and B compartments are separately analyzed. Most of upregulated genes in UNC0642-treated mESCs both in the A and B compartments are derepressed also in *Setdb1* KO mESCs. **d** Violin plot of log2 fold change of upregulated genes in *Setdb1* KO mESCs. Genes in A and B compartments are separately analyzed. Upregulated genes in *Setdb1* KO mESCs in the B compartments are more derepressed in UNC0642-treated mESCs than those genes in the A compartments. **e** Violin plot of log2 fold change of upregulated genes in UNC0642-treated *Setdb1* KO mESCs between *Setdb1* KO mESCs and UNC0642-treated *Setdb1* KO mESCs. Genes in A and B compartments are separately analyzed. Those genes in the B compartments are more upregulated by the UNC0642 treatment in *Setdb1* KO mESCs than those in the A compartments. P-value was calculated by student T-test. **f** Enrichment of H3K9me2 around genes identified in Fig. 4d in *G9a/GLP* DKO mESCs.

Supplementary Fig. 5

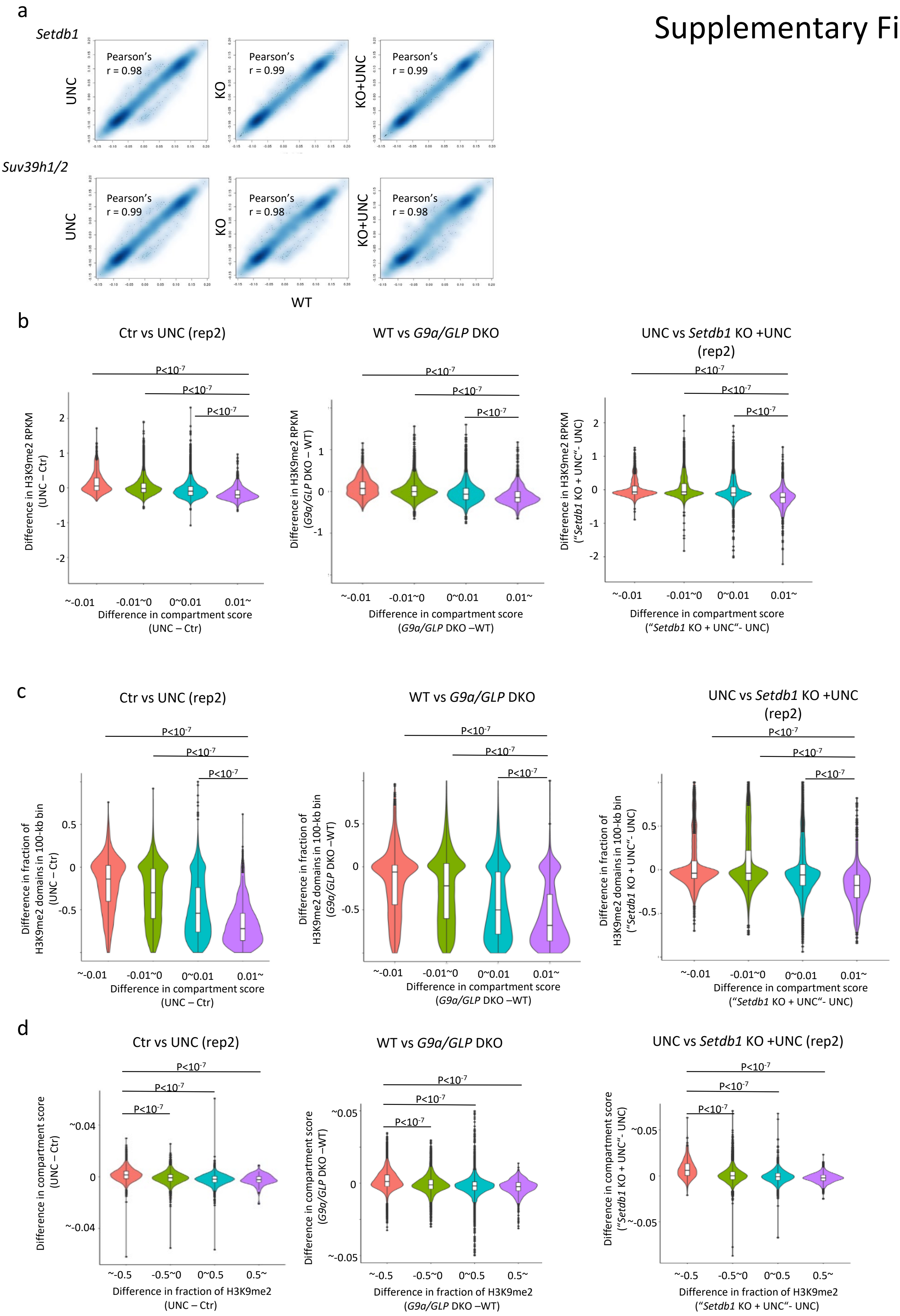

e

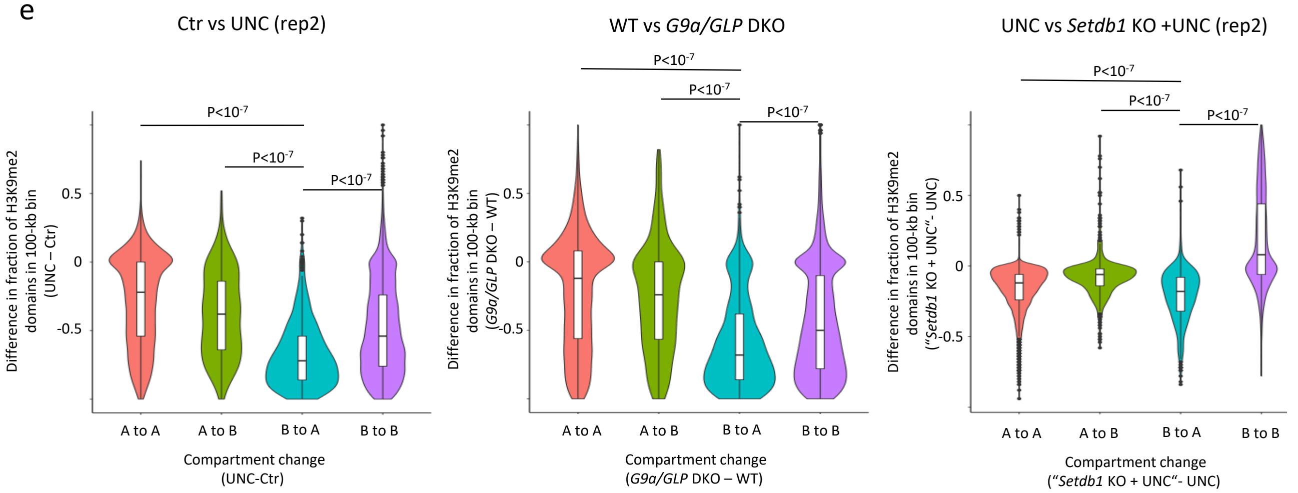
Ctr vs UNC (rep2)
WT vs G9a/GLP DKO
UNC vs Setdb1 KO +UNC (rep2)
P<10-7
Difference in fraction of H3K9me2 domains in 100-kb bin (UNC – Ctr)
Difference in fraction of H3K9me2 domains in 100-kb bin (G9a/GLP DKO – WT)
Difference in fraction of H3K9me2 domains in 100-kb bin ("Setdb1 KO + UNC"- UNC)
0.5
0
-0.5
A to A
A to B
B to A
B to B
Compartment change (UNC-Ctr)
Compartment change (G9a/GLP DKO – WT)
Compartment change ("Setdb1 KO + UNC"- UNC)

f

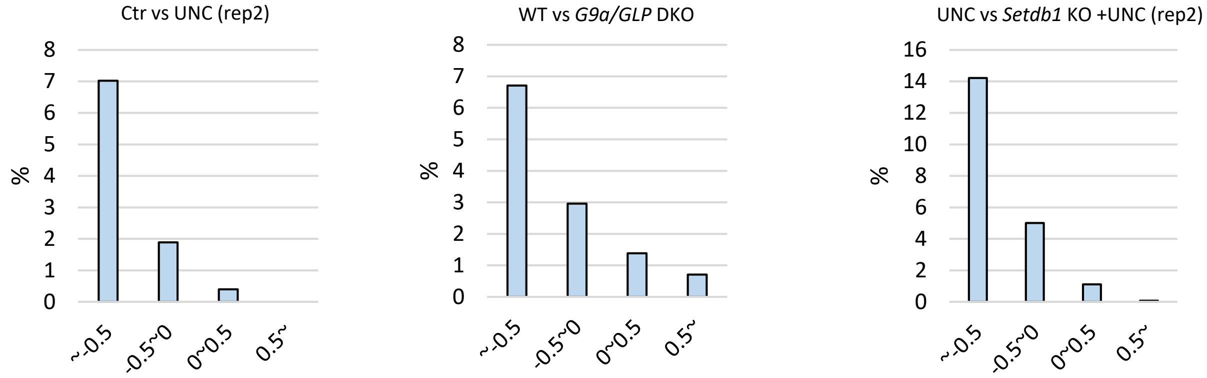
Ctr vs UNC (rep2)
WT vs G9a/GLP DKO
UNC vs Setdb1 KO +UNC (rep2)
%
~-0.5
-0.5~0
0~0.5
0.5~

g

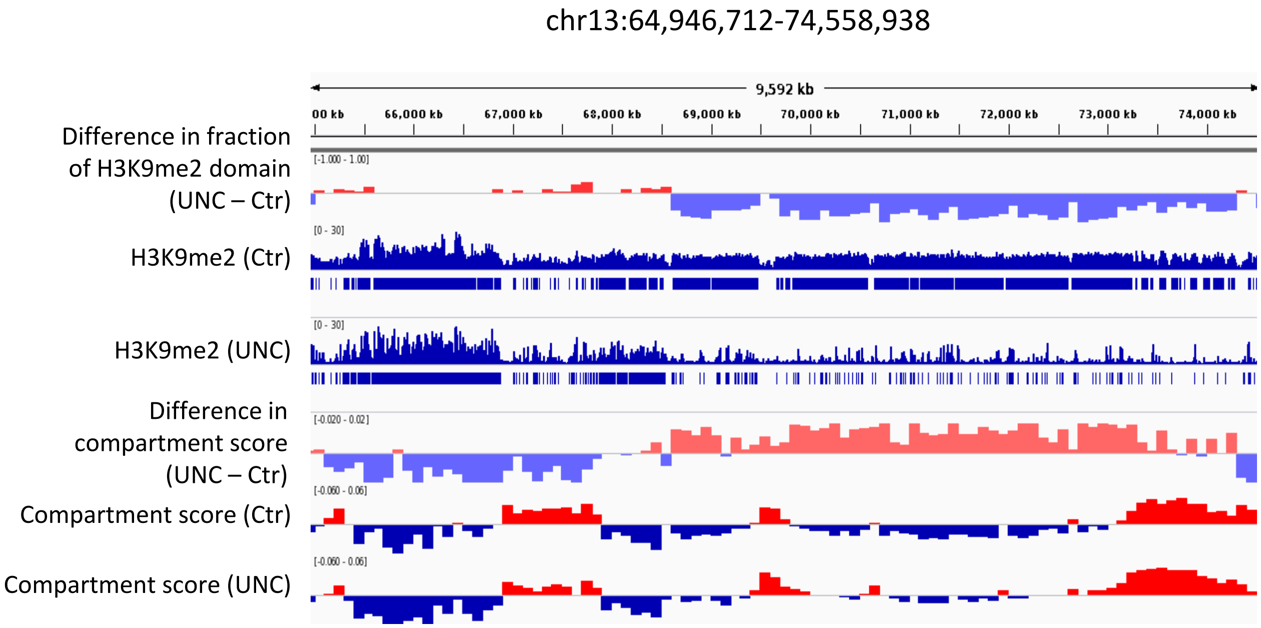
chr13:64,946,712-74,558,938
9,592 kb
66,000 kb
67,000 kb
68,000 kb
69,000 kb
70,000 kb
71,000 kb
72,000 kb
73,000 kb
74,000 kb
Difference in fraction of H3K9me2 domain (UNC – Ctr)
H3K9me2 (Ctr)
H3K9me2 (UNC)
Difference in compartment score (UNC – Ctr)
Compartment score (Ctr)
Compartment score (UNC)

Supplementary Fig. 5

a Scatter plot of compartment score between control cells and each condition. **b-f** Correlation between H3K9me2 reduction and increased compartment score related to Fig. 5C-G. Biological replicate and *G9a/GLP* DKO mESCs data are shown. (b) Violin plots showing differences in H3K9me2 RPKM in each 100-kb bin classified by a degree of compartment score changes in UNC0642-treated mESCs or *G9a/GLP* DKO mESCs. Increased compartment score was correlated with decreased H3K9me2. P-value was calculated by Tukey's test. Statistics comparison was only shown between the bins with ≧0.01 (0.01~) difference in compartment score and others. **c** Violin plots showing differences in H3K9me2 domain fraction in each 100-kb bin classified by a degree of compartment score changes in UNC0642-treated mESCs or *G9a/GLP* DKO mESCs. Increased compartment score was correlated with decreased H3K9me2 domain. P-value was calculated by Tukey's test. Statistics comparison was only shown between the bins with ≧0.01 (0.01~) difference in compartment score and others. **d** Violin plots showing changes of compartment scores in each 100-kb bin classified by a degree of changes in H3K9me2 domain fraction in UNC0642-treated mESCs or *G9a/GLP* DKO mESCs. Decreased H3K9me2 domain was correlated with increased compartment score. P-value was calculated by Tukey's test. Statistics comparison was only shown between the bins with <-0.5 (~-0.5) difference in fraction of H3K9me2 and others. **e** Violin plots showing changes of fraction of H3K9me2 domain in 100-kb bin classified by change of compartment pattern in UNC0642-treated mESCs or *G9a/GLP* DKO mESCs. B to A compartment change was correlated with decreased H3K9me2 domains. P-value was calculated by Tukey's test. Statistics comparison was only shown between the bins classified "B to A" and others. **f** Bar graph showing fraction of B to A compartment changes in each 100-kb bin classified by difference in H3K9me2 domain fraction in UNC0642-treated mESCs or *G9a/GLP* DKO mESCs. **g** Representative genomic region showing increased compartment score and decreased H3K9me2 in UNC0642-treated mESCs.

Western blot

Supplementary Fig. 1a, l

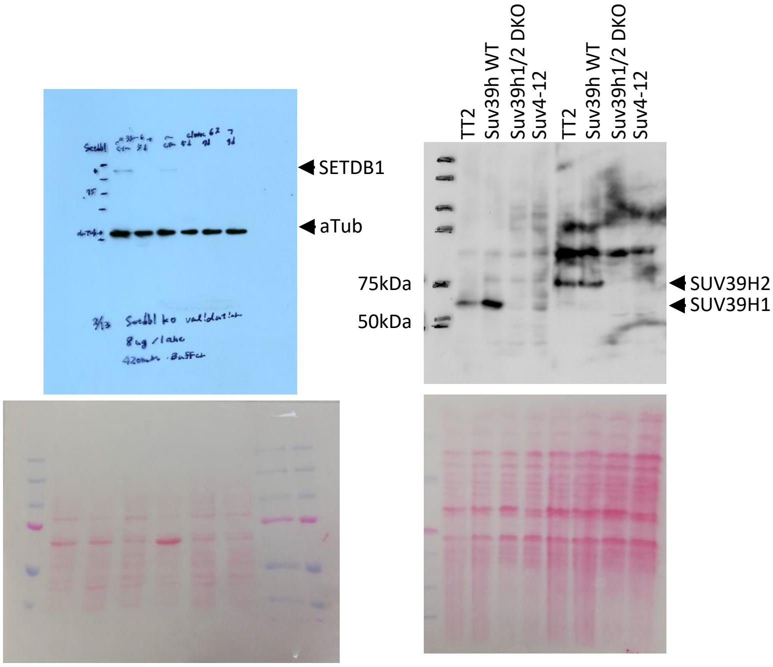

Supplementary Fig. 1c

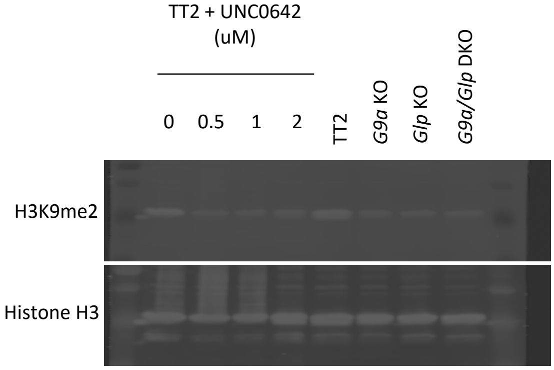

Supplementary Fig. 1m
SETDB1

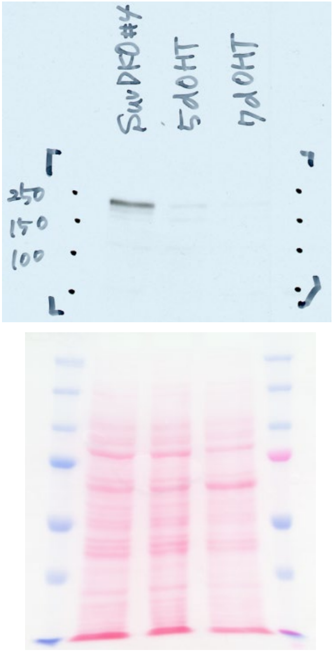

Supplementary Fig. 1m

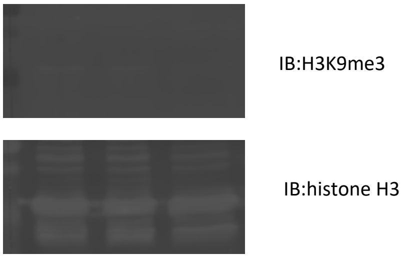

Supplementary Fig. 1n

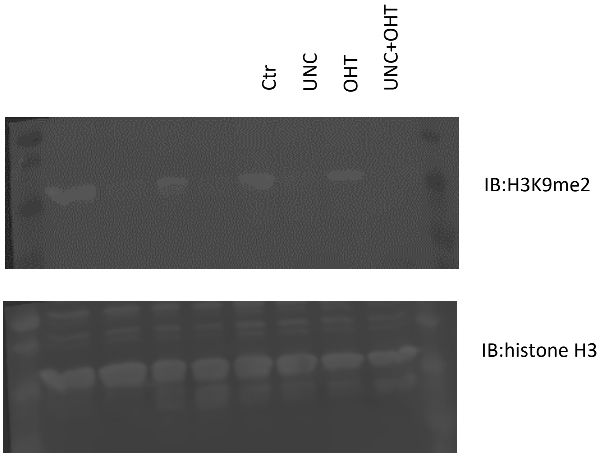

Supplementary Fig. 2f

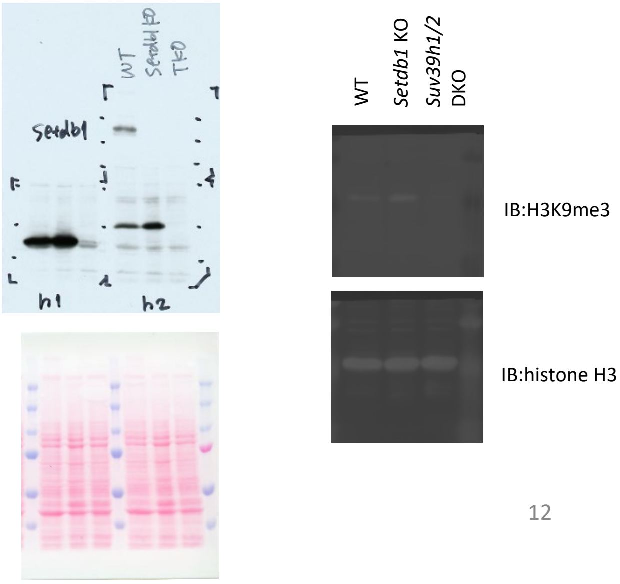

Western blot

Supplementary Fig. 2g

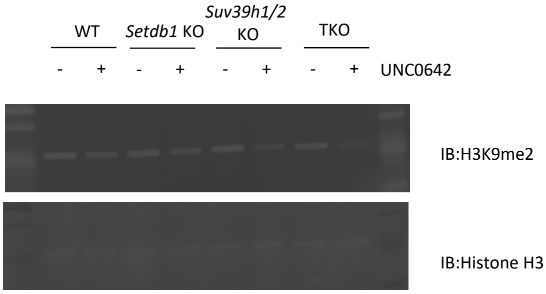

Supplementary Fig. 3a

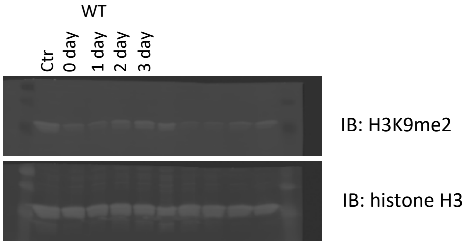

Supplementary Fig. 3c

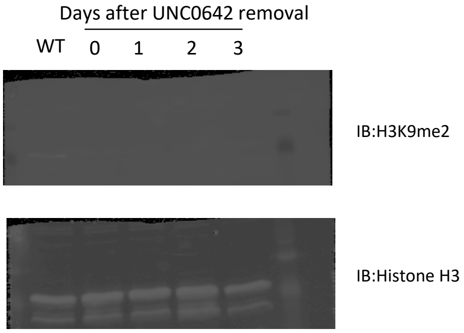

Supplementary Fig. 3d

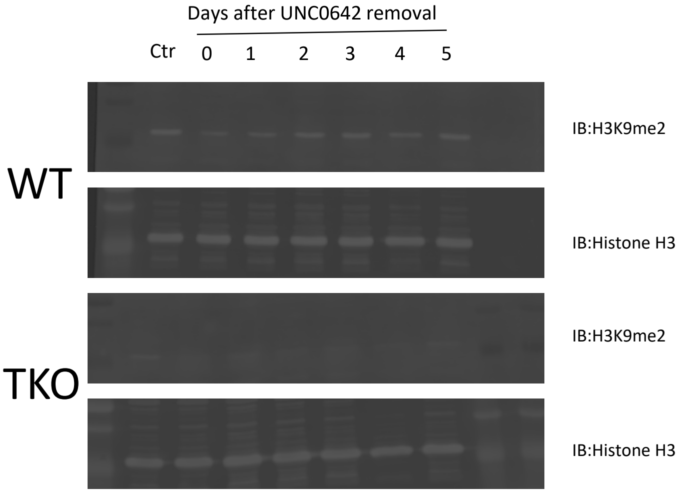

Supplementary Table 1: Correlation between replicate in RNA-seq, ChIP-seq, Hi-C seq

RNA-seq	Pearson's R (log2 RPM of genes)
WT mES	0.97
WTmES +UNC	0.97
Setdb1 KO mES	0.97
Setdb1 KO mES + UNC	0.96

ChIP-seq	Pearson's R (RPM in 100-kb bin)
WT mES	0.92
WT mES + UNC	0.96
Setdb1 KO mES	0.91
Setdb1 KO mES + UNC	0.95
Suv39h1/2 mES DKO	0.76
Suv39h1/2 DKO mES + UNC	0.89
G9a/GLP DKO mES	0.96
WT iMEF	0.86
Setdb1 KO iMEF	0.91
WT iMEF + UNC	0.89
Setdb1 KO iMEF + UNC	0.9

Hi-C	Pearson's R (Compartment score in 100-kb bin)
Setdb1 WT mESCs	0.98
Setdb1 WT mESCs + UNC	0.98
Setdb1 KO mESCs	0.98
Setdb1 KO mES + UNC	0.98
Suv39h1/2 WT mESCs	0.98
Suv39h1/2 WT mESCs + UNC	0.98
Suv39h1/2 DKO mESCs	0.98
Suv39h1/2 DKO mESCs + UNC	0.98

Supplementary Table 2: Primer list for ChIP-qPCR. qRT-PCR and genotyping

Target	Related figure	Aplication	Sequence Fw	Sequence Rv	Target regions
B compartment region 1	Fig. 1J and 2G	ChIP-qPCR	AGAGACAACATTGAGCCACCTT	ATGCAGGCTAAATGAAAGTAGACAG	chr14:80434190-80434292
B compartment region 2	Fig. 1J and 2G	ChIP-qPCR	TGCTCTGTCCTGATGTTGGA	TCCTTCGAGCTGAAAGCCTA	chr1:67712165-67712263
Nlrp4c	Fig. 4F	qRT-PCR	TTGAGGCTCTGAAACAGCAA	TCTTCCTCAGCCATCAGGAG	
Gm4971	Fig. 4F	qRT-PCR	TGCTCATCACTGTTCCTTGC	GAACGTGAGGACCATCCACT	
Trpd52l3	Fig. 4F	qRT-PCR	GTGGCGAACTCAAGAGGAAG	GTTGGAGGCCTGAACATCAT	

Supplementary Table 3: Oligonucleotide for gRNA vector construction

gRNA for iMEFs	
Suv39h2 exon 4 Upstream Fw	CACCTAAATAAGTTCCTATTACAG
Suv39h2 exon 4 Upstream Rv	AAACCTGTAATAGGAACTTATTTA
Suv39h2 exon 4 downstream Fw	CACCCACAGAATAGTACAGTAGTG
Suv39h2 exon 4 downstream Fw	AAACCACTACTGTACTATTCTGTG
Suv39h2 exon 3 Fw	CACCTCTACTACATTAACGAGTAC
Suv39h2 exon 3 Rv	AAACGTACTCGTTAATGTAGTAGA
Suv39h1 exon 4 Fw	CACCGCCGTGGATGCCGCTTATTA
Suv39h1 exon 4 Rv	AAACTAATAAGCGGCATCCACGGC

gRNA for mESCs	
Suv39h1 exon 3 Upstream Fw	CACCATTACCTGGTTAAGTGGCGT
Suv39h1 exon 3 Upstream Rv	AAACACGCCACTTAACCAGGTAAT
Suv39h1 exon 3 Downstream Fw	CACCTGTGGGAGAGGTGAGCATAT
Suv39h1 exon 3 Downstream Rv	AAACGATATGCTCACCTCTCCCACA
Suv39h2 exon 3 Fw	CACCTCTACTACATTAACGAGTAC
Suv39h2 exon 3 Rv	AAACGTACTCGTTAATGTAGTAGA
Suv39h2 exon 4 Fw	CACCACAGTGGATGCAGCTCGATA
Suv39h2 exon 4 Rv	AAACTATCGAGCTGCATCCACTGT

Supplementary Table 4 Sumary of NGS data set

ChIP-seq sample	Target	Replicate	Cell line	Paper related to cell line	Antibody	Application	Read number
WT mESCs (TT2)	H3K9me2	Rep1	TT2		6D11	N-ChIP-seq	61793662
Setdb1 WT mESCs	H3K9me2	Rep1	#33-6 *Setdb1* conditional KO	Matsui et al., Nature, 2011	6D11	N-ChIP-seq	153780494
Setdb1 WT mESCs rep2	H3K9me2	Rep2	#33-6 *Setdb1* conditional KO	Matsui et al., Nature, 2011	6D11	N-ChIP-seq	96410938
Setdb1 WT mESCs + UNC	H3K9me2	Rep1	#33-6 *Setdb1* conditional KO	Matsui et al., Nature, 2011	6D11	N-ChIP-seq	79680206
Setdb1 WT mESCs + UNC rep2	H3K9me2	Rep2	#33-6 *Setdb1* conditional KO	Matsui et al., Nature, 2011	6D11	N-ChIP-seq	52888702
Setdb1 KO mESCs	H3K9me2	Rep1	#33-6 *Setdb1* conditional KO	Matsui et al., Nature, 2011	6D11	N-ChIP-seq	56617514
Setdb1 KO mESCs rep2	H3K9me2	Rep2	#33-6 *Setdb1* conditional KO	Matsui et al., Nature, 2011	6D11	N-ChIP-seq	105555816
Setdb1 KO mESCs + UNC	H3K9me2	Rep1	#33-6 *Setdb1* conditional KO	Matsui et al., Nature, 2011	6D11	N-ChIP-seq	57201694
Setdb1 KO mESCs + UNC rep2	H3K9me2	Rep2	#33-6 *Setdb1* conditional KO	Matsui et al., Nature, 2011	6D11	N-ChIP-seq	75057672
Suv39h1/2 WT mESCs	H3K9me2	Rep1	ADF1-26 gifted from T.J.	Lehnertz et al., Curr. Biol, 2003	6D11	N-ChIP-seq	62630690
Suv39h1/2 WT mESCs + UNC	H3K9me2	Rep1	ADF1-26 gifted from T.J.	Lehnertz et al., Curr. Biol, 2003	6D11	N-ChIP-seq	71784846
Suv39h1/2 DKO mESCs	H3K9me2	Rep1	ADF157 gifted from T. J.	Lehnertz et al., Curr. Biol, 2003	6D11	N-ChIP-seq	71688486
Suv39h1/2 DKO mESCs rep2	H3K9me2	Rep2	#4 Suv39h1/2 DKO	This study	6D11	N-ChIP-seq	41360106
Suv39h1/2 DKO mESCs *+ UNC*	H3K9me2	Rep1	ADF157 gifted from T. J.	Lehnertz et al., Curr. Biol, 2003	6D11	N-ChIP-seq	72532350
Suv39h1/2 DKO mESCs *+ UNC* rep2	H3K9me2	Rep2	#4 Suv39h1/2 DKO	This study	6D11	N-ChIP-seq	42477064
G9a/GLP DKO mESCs	H3K9me2	Rep1	#248-6 G9a/DLP DKO	Tachibana et al., 2005	6D11	N-ChIP-seq	64207724
G9a/GLP DKO mESCs rep2	H3K9me2	Rep2	#248-6 G9a/DLP DKO	Tachibana et al., 2005	6D11	N-ChIP-seq	90665156
Setdb1 WT iMEFs	H3K9me2	Rep1		Kato et al., Nat. Com., 2018	6D11	N-ChIP-seq	54537876
Setdb1 WT iMEFs + UNC	H3K9me2	Rep1		Kato et al., Nat. Com., 2018	6D11	N-ChIP-seq	63064272
Setdb1 KO iMEFs	H3K9me2	Rep1	3-12#13	Kato et al., Nat. Com., 2018	6D11	N-ChIP-seq	69219922
Setdb1 KO iMEFs + UNC	H3K9me2	Rep1	3-12#13	Kato et al., Nat. Com., 2018	6D11	N-ChIP-seq	39855692
Setdb1 WT iMEFs	H3K9me2	Rep1			6D11	N-ChIP-seq	76106976
Setdb1 WT iMEFs + UNC	H3K9me2	Rep1			6D11	N-ChIP-seq	62315274
Setdb1 KO iMEFs	H3K9me2	Rep1	3-12#13	Kato et al., Nat. Com., 2018	6D11	N-ChIP-seq	47725926
Setdb1 KO iMEFs + UNC	H3K9me2	Rep1	3-12#13	Kato et al., Nat. Com., 2018	6D11	N-ChIP-seq	55745836
0h after UNC0642 removal	H3K9me2	Rep1	TT2		6D11	N-ChIP-seq	67264338
24h after UNC0642 removal	H3K9me2	Rep1	TT2		6D11	N-ChIP-seq	41814996
32h after UNC0642 removal	H3K9me2	Rep1	TT2		6D11	N-ChIP-seq	51067302
40h after UNC0642 removal	H3K9me2	Rep1	TT2		6D11	N-ChIP-seq	108581210
48h after UNC0642 removal	H3K9me2	Rep1	TT2		6D11	N-ChIP-seq	93862706
56h after UNC0642 removal	H3K9me2	Rep1	TT2		6D11	N-ChIP-seq	55850632
64h after UNC0642 removal	H3K9me2	Rep1	TT2		6D11	N-ChIP-seq	42511934
72h after UNC0642 removal	H3K9me2	Rep1	TT2		6D11	N-ChIP-seq	50751830
Setdb1 WT mESC	H3K9me3	Rep1	#33-6 *Setdb1* conditional KO	This study	2F3	N-ChIP-seq	47374932
Setdb1 WT mESC + UNC	H3K9me3	Rep1	#33-6 *Setdb1* conditional KO	This study	2F3	N-ChIP-seq	48004050
Setdb1 KO mESCs	H3K9me3	Rep1	#33-6 *Setdb1* conditional KO	This study	2F3	N-ChIP-seq	48669244
Setdb1 KO mESCs + UNC	H3K9me3	Rep1	#33-6 *Setdb1* conditional KO	This study	2F3	N-ChIP-seq	54642090
TT2	Input	Rep1	TT2		-	N-ChIP-seq	75571718
Setdb1 WT iMEFs	Input	Rep1			-	N-ChIP-seq	85043054

RNA-seq sample	Target	Replidate	Cell line	Paper related to cell line	Antibody	Application	Read number
Setdb1 WT mESCs	poly A RNA	Rep1	#33-6 *Setdb1* conditional KO	Matsui et al., Nature, 2011	-	RNA-seq	26080580
Setdb1 WT mESCs + UNC	poly A RNA	Rep1	#33-6 *Setdb1* conditional KO	Matsui et al., Nature, 2011	-	RNA-seq	51313972
Setdb1 KO mESCs	poly A RNA	Rep1	#33-6 *Setdb1* conditional KO	Matsui et al., Nature, 2011	-	RNA-seq	61755616
Setdb1 KO *mESCs + UNC*	poly A RNA	Rep1	#33-6 *Setdb1* conditional KO	Matsui et al., Nature, 2011	-	RNA-seq	27741886
Setdb1 WT mESCs rep2	poly A RNA	Rep2	#33-6 *Setdb1* conditional KO	Matsui et al., Nature, 2011	-	RNA-seq	55777800
Setdb1 WT mESCs + UNC rep2	poly A RNA	Rep2	#33-6 *Setdb1* conditional KO	Matsui et al., Nature, 2011	-	RNA-seq	52318630
Setdb1 KO mESCs rep2	poly A RNA	Rep2	#33-6 *Setdb1* conditional KO	Matsui et al., Nature, 2011	-	RNA-seq	37100416
Setdb1 KO *mESCs + UNC rep2*	poly A RNA	Rep2	#33-6 *Setdb1* conditional KO	Matsui et al., Nature, 2011	-	RNA-seq	60061236

Hi-C seq sample	Restriction enzyr	Replidate	Cell line	Paper related to cell line	Antibody	Application	Read number
Setdb1 WT mESCs	DpnII	Rep1	#33-6 *Setdb1* conditional KO	Matsui et al., Nature, 2011	-	Hi-C	115,102,613
Setdb1 WT mESCs + UNC	DpnII	Rep1	#33-6 *Setdb1* conditional KO	Matsui et al., Nature, 2011	-	Hi-C	124,974,183
Setdb1 KO mESCs	DpnII	Rep1	#33-6 *Setdb1* conditional KO	Matsui et al., Nature, 2011	-	Hi-C	137,683,964
Setdb1 KO mESCs + UNC	DpnII	Rep1	#33-6 *Setdb1* conditional KO	Matsui et al., Nature, 2011	-	Hi-C	122,801,633
Suv39h1/2 WT mESCs	DpnII	Rep1	ADF1-26 gifted from T.J.	Lehnertz et al., Curr. Biol, 2003	-	Hi-C	116,829,424
Suv39h1/2 WT mESCs + UNC	DpnII	Rep1	ADF1-26 gifted from T.J.	Lehnertz et al., Curr. Biol, 2003	-	Hi-C	119,212,789
Suv39h1/2 DKO mESCs	DpnII	Rep1	ADF157 gifted from T. J.	Lehnertz et al., Curr. Biol, 2003	-	Hi-C	79,689,521
Suv39h1/2 DKO mESCs *+ UNC*	DpnII	Rep1	ADF157 gifted from T. J.	Lehnertz et al., Curr. Biol, 2003	-	Hi-C	89,177,357
Setdb1 WT mESCs	DpnII	Rep2	#33-6 *Setdb1* conditional KO	Matsui et al., Nature, 2011	-	Hi-C	105,914,121
Setdb1 WT mESCs + UNC	DpnII	Rep2	#33-6 *Setdb1* conditional KO	Matsui et al., Nature, 2011	-	Hi-C	100,816,025
Setdb1 KO mESCs	DpnII	Rep2	#33-6 *Setdb1* conditional KO	Matsui et al., Nature, 2011	-	Hi-C	103,504,579
Setdb1 KO mESCs + UNC	DpnII	Rep2	#33-6 *Setdb1* conditional KO	Matsui et al., Nature, 2011	-	Hi-C	101,971,299
Suv39h1/2 WT mESCs	DpnII	Rep2	ADF1-26 gifted from T.J.	Lehnertz et al., Curr. Biol, 2003	-	Hi-C	100,930,051
Suv39h1/2 WT mESCs + UNC	DpnII	Rep2	ADF1-26 gifted from T.J.	Lehnertz et al., Curr. Biol, 2003	-	Hi-C	96,578,569
Suv39h1/2 DKO mESCs	DpnII	Rep2	ADF157 gifted from T. J.	Lehnertz et al., Curr. Biol, 2003	-	Hi-C	137,647,002
Suv39h1/2 DKO mESCs *+ UNC*	DpnII	Rep2	ADF157 gifted from T. J.	Lehnertz et al., Curr. Biol, 2003	-	Hi-C	97,023,584
WT *iMEFs*	DpnII	Rep1			-	Hi-C	108758483
