# Supplementary material for: Regulation of mammalian 3D genome organization and histone H3K9 dimethylation by H3K9 methyltransferases
Source: Commun Biol. 2021 May 13;4:571. doi: 10.1038/s42003-021-02089-y (PMC8119675; doi:10.1038/s42003-021-02089-y)
Supplement: Supplementary file 3 — Description of Additional Supplementary File [file 42003_2021_2089_MOESM3_ESM.pdf]

Description of additional supplementary file

File name: Supplementary Data 1

Description: Source data for all main figures
