# Supplementary material for: Regulation of mammalian 3D genome organization and histone H3K9 dimethylation by H3K9 methyltransferases
Source: Commun Biol. 2021 May 13;4:571. doi: 10.1038/s42003-021-02089-y (PMC8119675; doi:10.1038/s42003-021-02089-y)
Supplement: Supplementary file 5 — Reporting Summary [file 42003_2021_2089_MOESM5_ESM.pdf]

# nature research

Corresponding author(s): Kei Fukuda and Yoichi Shinkai

Last updated by author(s): Mar 20, 2021

# Reporting Summary

Nature Research wishes to improve the reproducibility of the work that we publish. This form provides structure for consistency and transparency in reporting. For further information on Nature Research policies, see our Editorial Policies and the Editorial Policy Checklist.

## Statistics

For all statistical analyses, confirm that the following items are present in the figure legend, table legend, main text, or Methods section.

| n/a | Confirmed | |
|---|---|---|
| ☐ | ☒ | The exact sample size (*n*) for each experimental group/condition, given as a discrete number and unit of measurement |
| ☐ | ☒ | A statement on whether measurements were taken from distinct samples or whether the same sample was measured repeatedly |
| ☐ | ☒ | The statistical test(s) used AND whether they are one- or two-sided<br>*Only common tests should be described solely by name; describe more complex techniques in the Methods section.* |
| ☒ | ☐ | A description of all covariates tested |
| ☐ | ☒ | A description of any assumptions or corrections, such as tests of normality and adjustment for multiple comparisons |
| ☐ | ☒ | A full description of the statistical parameters including central tendency (e.g. means) or other basic estimates (e.g. regression coefficient) AND variation (e.g. standard deviation) or associated estimates of uncertainty (e.g. confidence intervals) |
| ☐ | ☒ | For null hypothesis testing, the test statistic (e.g. *F*, *t*, *r*) with confidence intervals, effect sizes, degrees of freedom and *P* value noted<br>*Give P values as exact values whenever suitable.* |
| ☒ | ☐ | For Bayesian analysis, information on the choice of priors and Markov chain Monte Carlo settings |
| ☒ | ☐ | For hierarchical and complex designs, identification of the appropriate level for tests and full reporting of outcomes |
| ☒ | ☐ | Estimates of effect sizes (e.g. Cohen's *d*, Pearson's *r*), indicating how they were calculated |

*Our web collection on statistics for biologists contains articles on many of the points above.*

## Software and code

Policy information about availability of computer code

| | |
|---|---|
| Data collection | NGS (ChIP-seq, RNA-seq, Hi-C): HiSeq X |
| Data analysis | Trim_galore v0.3.7, bowtie v0.12.6, Tophat v2.1.1, DESeq2 v1.20.0, hiddendomain, IGV v2.3.49, R v3.5, deeptools v2.5.4, 4DN Hi-C pipeline v43, samtools v0.1.18, Homer v4.8.3 |

For manuscripts utilizing custom algorithms or software that are central to the research but not yet described in published literature, software must be made available to editors and reviewers. We strongly encourage code deposition in a community repository (e.g. GitHub). See the Nature Research guidelines for submitting code & software for further information.

## Data

Policy information about availability of data

All manuscripts must include a data availability statement. This statement should provide the following information, where applicable:

- Accession codes, unique identifiers, or web links for publicly available datasets
- A list of figures that have associated raw data
- A description of any restrictions on data availability

ChIP, RNA and Hi-C seq data have been submitted in GEO under accession number GSE169106. The mass spectrometry proteomics data have been deposited to the ProteomeXchange Consortium via the PRIDE [1] partner repository with the dataset identifier PXD024756 and 10.6019/PXD024756.

# Field-specific reporting

Please select the one below that is the best fit for your research. If you are not sure, read the appropriate sections before making your selection.

☒ Life sciences ☐ Behavioural & social sciences ☐ Ecological, evolutionary & environmental sciences

For a reference copy of the document with all sections, see nature.com/documents/nr-reporting-summary-flat.pdf

# Life sciences study design

All studies must disclose on these points even when the disclosure is negative.

| | |
|---|---|
| Sample size | No sample-size calculations were performed. |
| Data exclusions | No data exclusion was performed in this study |
| Replication | All attempts of replication were successful. The exact number of times each experiment was performed is stated in the corresponding figure legend. |
| Randomization | No randomization was performed in this study |
| Blinding | No blinding was performed in this study |

# Reporting for specific materials, systems and methods

We require information from authors about some types of materials, experimental systems and methods used in many studies. Here, indicate whether each material, system or method listed is relevant to your study. If you are not sure if a list item applies to your research, read the appropriate section before selecting a response.

## Materials & experimental systems

| n/a | Involved in the study | |
|---|---|---|
| ☐ | ☒ | Antibodies |
| ☐ | ☒ | Eukaryotic cell lines |
| ☒ | ☐ | Palaeontology and archaeology |
| ☒ | ☐ | Animals and other organisms |
| ☒ | ☐ | Human research participants |
| ☒ | ☐ | Clinical data |
| ☒ | ☐ | Dual use research of concern |

## Methods

| n/a | Involved in the study | |
|---|---|---|
| ☐ | ☒ | ChIP-seq |
| ☒ | ☐ | Flow cytometry |
| ☒ | ☐ | MRI-based neuroimaging |

## Antibodies

| | |
|---|---|
| Antibodies used | antibodies specific for histone H3 (07-690, EMD Millipore), H3K9me3 (2F3), H3K9me2 (6D11 and39754, Active Motif), histone H3 (96C10, Cell Signaling), SETDB1 (Cp10377, Cell Applications), SUV39H1 (#8729, CST), SUV39H2 (LS-116360, LSBio), G9a (A8620A) and GLP (B0422B) were used as primary antibody. |
| Validation | Specificity of antibodies for histone modifications were validated by the removal or inhitibition of the corresponding histone methyltransferase. SETDB1, SUV39H1/2, G9a/GLP antibodies were validated by knockout of the corresponding gene. |

## Eukaryotic cell lines

Policy information about cell lines

| | |
|---|---|
| Cell line source(s) | Setdb1 conditional knockout mESCs, Setdb1 KO iMEFs, G9a KO mESCs, Glp KO mESCs, G9a/GLP DKO mESCs were previously established in our lab. Setdb1/Suv39h1/2 TKO mESCs and iMEFs were established in this study. Suv39h1/2 DKO mESCs and iMEFs were kindly gifted from Thomas Jenuwein (Lehnerts et al., Curr. Biol., 2003, Lachner et al., Nature, 2001). |
| Authentication | No cell authentication was performed |
| Mycoplasma contamination | the cell lines were not tested for mycoplasma contamination |
| Commonly misidentified lines (See ICLAC register) | No commonly misidentified cell lines were used in this study. |

# ChIP-seq

## Data deposition

☒ Confirm that both raw and final processed data have been deposited in a public database such as GEO.

☒ Confirm that you have deposited or provided access to graph files (e.g. BED files) for the called peaks.

| | |
|---|---|
| Data access links<br>*May remain private before publication.* | All genome profiling data have now been deposited in the Gene Expression Omnibus (GEO) repository under accession codes GSE169106 |
| Files in database submission | Bigwig files for all ChIP-seq data |
| Genome browser session<br>(e.g. UCSC) | Bigwigs available to download for browsing in genome broser of choice. |

## Methodology

| | |
|---|---|
| Replicates | H3K9me2 ChIP-seq in WT, G9a/GLP DKO, Setdb1 KO and Suv39h1/2 DKO mESCs treated or untreated with UNC0642 were conducted in two biological experiments. H3K9me2 ChIP-seq in WT and Setdb1 KO iMEFs treated or untreated with UNC0642 were conducted two biological experiments. H3K9me2 ChIP-seq after UNC0642 removal were conducted one biological expriments. H3K9me3 ChIP-seq in WT, Setdb1 KO mESCs treated or untreated with UNC0642 were conducted in one biological experiments. RNA-seq in WT and Setdb1 KO mESCs treated with or without UNC0642 were conducted two biological experiments. Hi-C in WT, Setdb1 KO and Suv39h1/2 DKO mESCs treated with or without UNC0642 were conducted two biological experiments. |
| Sequencing depth | All NGS were performed as 150-bp paired-ended. On average each ChIP-seq sample contained 65M reads with min 39M and max 153M reads. On average each RNA-seq sample contained 46M reads with min 26M and max 61M reads. On average each Hi-C sample contained 109M read-pairs with min 79M and 137M read-pairs. |
| Antibodies | H3K9me2 (6D11) and H3K9me3 (2F3) were used for ChIP-seq. |
| Peak calling parameters | Adaptor sequences in reads were trimmed using Trim Galore version 0.3.7. Then trimmed reads were aligned to the mm10 genome build using bowtie version 0.12.7 with default parameters. Duplicated reads were removed using samtools version 0.1.18. Enriched or depleted status of H3K9me2 in each 2kb was determined by Hiddendomains and continuous H3K9me2 enriched 2kb regions was connected as H3K9me2 domain with some modifications: The option of Bin size and max.read.count was 2,000 bp and 150, respectively. Read number in each bin was normalized by the following formula to adjust difference in read number among samples. Normalized read number = read number x 30,000,000 / total read number. |
| Data quality | We did not identify peaks. |
| Software | Trim_galore v0.3.7, bowtie v0.12.6, Tophat v2.1.1, DESeq2 v1.20.0, hiddendomain, IGV v2.3.49, R v3.5, deeptools v2.5.4, 4DN Hi-C pipeline v43, samtools v0.1.18, Homer v4.8.3 |
